# Light‐Driven Continual Oscillatory Rocking of a Polymer Film

**DOI:** 10.1002/open.202000237

**Published:** 2020-11-06

**Authors:** Marina Pilz da Cunha, Akhil R. Peeketi, Adithya Ramgopal, Ratna K. Annabattula, Albert P. H. J. Schenning

**Affiliations:** ^1^ Laboratory of Stimuli-responsive Functional Materials & Devices, Department of Chemical Engineering and Chemistry Eindhoven University of Technology 5600 MB Eindhoven The Netherlands; ^2^ Stimuli-Responsive Systems Laboratory, Department of Mechanical Engineering Indian Institute of Technology Madras (IITM) 600036 Chennai India

**Keywords:** light-driven oscillation, polymers, azo compounds, sustained motion, light-driven liquid crystal actuators

## Abstract

Achieving oscillatory motion in polymers without requiring on/off switching of stimuli is a current challenge. Hereby, a free‐standing liquid crystal polymer (LCP) is demonstrated to undergo a sustained oscillatory motion when triggered by light, moving back and forth, resembling the motion of a rocking‐chair. Two polymer films having different azobenzene photo‐switches have been studied, revealing photoswitch requirements as well as illumination conditions necessary to sustain oscillations. The motion presented here shows how feedback loops involving light‐triggered actuation, self‐shadowing and a shifting center of gravity can be utilized to achieve self‐sustained motion in free‐standing polymers.

## Graphical abstract

**Sustained oscillations** in stimuli‐responsive actuators will increase the autonomy of future devices. Here, a liquid crystal polymer is shown to undergo continuous light‐driven oscillation without requiring on/off switching of the light source.

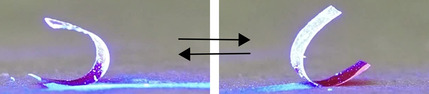

Stimuli‐responsive liquid crystal polymers (LCPs) have become popular materials in the development of untethered soft actuators.[^1^, ^2^] The use of light to control motion has received particular attention due to its ease of addressability and tunability as well as high spatio‐temporal accuracy.[^3^, ^4^] Light‐triggered motion in LCPs containing photoswitches, has expanded from simple bending and elongation/contraction towards more complex motions[^5^, ^6^] resulting in, for example, light‐driven soft robots with advanced abilities such as locomotion or cargo handling.[^7^, ^8^, ^9^] To increase locomotion speed and facilitate control over the actuation of such robots, strategies to achieve constant oscillatory motion of LCPs without requiring turning on/off the stimulus are of interest. Even though examples in the literature show liquid crystal (LC) films containing photothermal dyes can oscillate upon illumination when clamped on one side,[^10^, ^11^, ^12^, ^13^, ^14^, ^15^] the development of autonomous light‐driven soft robots calls for films that can undergo oscillation when placed freely on a surface. Thermally‐driven actuation of a free‐standing single liquid crystal network (LCN) has been previously presented,^[16]^ but light‐driven oscillations remain unreported. Similar rocking motion of curled polymers has been previously reported for light‐driven polymer bilayers.[^17^, ^18^, ^19^]

Here, a free‐standing LCP is shown to undergo light‐triggered oscillatory motion when illuminated at an oblique angle, Figure 1A. In its rest/non‐illuminated state, the film is curled, forming a “U” shape, and upon illumination, it rocks between two extreme states, towards and away from the light source. The motion is sustained until the light is turned off. To uncover the mechanism and conditions necessary to sustain this motion, two identical liquid crystal networks (LCN)s with differing azobenzene photoswitches, Figure 1B, are investigated. Both photoswitches trigger actuation through photothermal effects in which heat released through light absorption dissipates in the network and actuation happens as a result of anisotropic thermal expansions of the network.^[20]^

**Figure 1:**
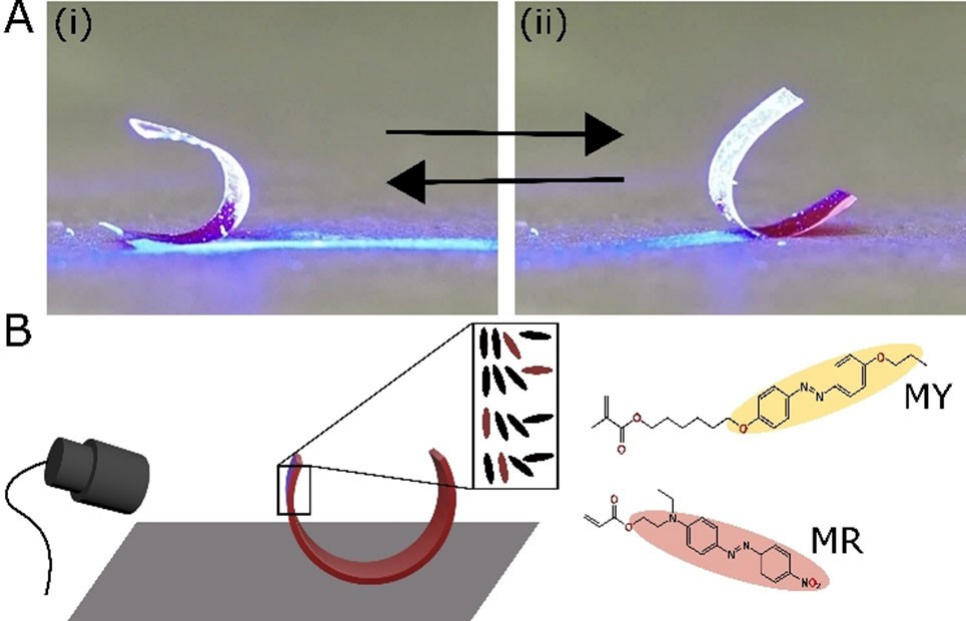
**A**. Photographs of the light‐driven LCP oscillator (containing photoswitch MR) as it rocks back and forth, towards and away from the light source (directed from the left). The polymer film is 1.5 cm in length. **B**. Schematic of the splay aligned LCN oscillator containing the photothermal azobenzene chromophores (MR and MY). For oscillatory actuation to take place, the blue light source is positioned at an angle as to irradiate only the frontal area of the film when in the position depicted in the schematic.

The LCNs studied have been reported before,^[21]^ are 20 μm in thickness and have a splay molecular alignment, in which at one surface the molecules are oriented perpendicular and at the other parallel to the film surface, see Figure 1B. The curled rest state is inherent from anisotropic thermal expansions after polymerization at elevated temperatures.^[16]^ The dependency of oscillations on the nature of the azobenzene photoswitch is investigated by comparing the actuation of two LCNs containing either azobenzene derivative MR or MY, Figure 1B. When the free‐standing U shaped samples are illuminated as shown in Figure 1B, LCNs containing azobenzene MY, do not oscillate but undergo an unbending deformation, causing both sides of the film to actuate towards the surface, at a similar unbending angle, Figure 2A, and return to the curled rest state once the light is turned off. Light‐driven unbending is caused by an increase in the film's temperature when the azobenzene photoswitches absorb incident light and release heat.^[21]^ The constructive effect of the anisotropic thermal expansions in the splay aligned network results in the unbending actuation of the U shaped film: when heated, the homeotropic side of the film (inside of the U shape) expands while the planarly aligned surface on the outside contracts in the direction parallel to the film length.^[22]^ On the other hand, films containing azobenzene MR initiate a rocking motion, tipping from one side (i) to the other (ii), Figure 2A and Movie S1.

**Figure 2:**
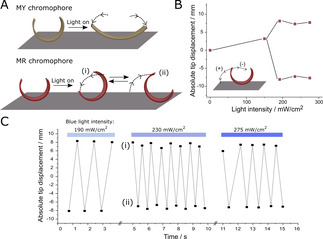
Characterization of oscillatory motion. **A**. Schematic showing the actuation of the two different light‐responsive films containing different chromophores, MR and MY. Upon illumination, from an oblique angle as shown in Figure 1B, the film containing azobenzene MY does not initiate oscillation, but both film tips bend towards the surface and remain in this position until illumination is stopped. On the other hand, films containing azobenzene MR initiate an oscillatory motion, rocking forward (i) and backwards (ii). **B**. Plot depicting the light intensity regime in which oscillatory motion takes place. Below this intensity (∼200 mW/cm^2^), the film only bends without displaying oscillatory motion. **C**. Plot displaying the oscillating motion over time at three different light intensities. The film rocks between two extreme states, (i) and (ii). The two extreme positions of the oscillator during oscillation: (i) and (ii) represent the positions shown by the data points in A.

The rocking motion observed in MR‐containing films was found to only happen when the illumination intensity was above a threshold, ∼200 mW/cm^2^, Figure 2B. Below this intensity, only the illuminated section of the film actuates by unbending towards the surface, but no rocking motion is observed. By tracking the position of the frontal section of the film during illumination with intensities above the threshold, both the frequency and amplitude of oscillations at three different light intensities can be visualized, Figure 2C. The plot shows that the amplitude of each oscillation does not significantly vary between the three different intensities, the motion always follows an oscillation between state (i) in which the frontal section is closer to the surface and (ii) in which it is furthest, see Figure 2C. The frequency of oscillations however do vary, from 1.7 Hz at 190 mW/cm^2^, to 3.1 Hz at 230 mW/cm^2^, and 2.6 Hz at 275 mW/cm^2^.

To understand the lack of oscillations from films containing azobenzene MY, the light absorbance profiles of both films were studied, Figure 3A. Even though both chromophores are doped in identical LC networks with the same molar concentration (2 mol %), the absorbance profile of films containing azobenzene MR is clearly higher. The direct impact of differing light absorbances in the actuation is observed when the two LCPs are clamped and placed in parallel (behind each other) for illumination, Figure 3B. In this scenario, light must first penetrate through the first film in order to reach the second. The lack of actuation from the rear film when MR‐films are placed in front reveals that the high absorbance of MR chromophore leads to a shadowing effect: the first film absorbs most incident light, shading the second film. When the scenario is switched, and MY films are placed in front, both the first and the second film actuate. This observation agrees with the motion observed Figure 2A: when freestanding and illuminated at an oblique angle, both frontal and rear sections of MY films actuate. The effective shadowing effect observed in Figure 3B, is also occurring when freestanding MR films are illuminated, so that the frontal section of the film shades the rear.

**Figure 3:**
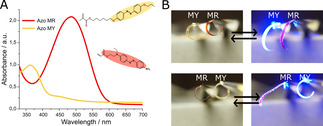
**A**. Absorbance plot for two splay aligned LCPs containing the different dyes. The absorbance of MR film is much higher, absorbing nearly all incident blue light (455 nm). **B**. Photographs of two light‐sensitive LCPs placed in series. The two films contain different photoswitches, MR and MY at the same concentration (2 mol %) of light sensitive molecule. When blue light is incident from the left both films actuate only if the film containing photoswitch MY is placed at the front. When MR is placed in front, the MY film behind does not actuate.

Based on these findings, we propose a mechanism for the oscillatory motion observed in films doped with azobenzene MR, Figure 4. The mechanism is rooted in self‐shadowing, so that when the nearest section of the film is illuminated, it shades the back. When illuminated, the front section of the film unbends, descending towards the surface, shifting the center of mass, causing the film to tip toward the light source, step (i) in Figure 4. When the film tips forward, it brings the front section out of the light beam and causes the back of the film to be exposed. This rear section then actuates by unbending towards the surface, step (ii), once again shifting the film's center of gravity, and causing the film to tip back. An additional contribution to the same backwards tipping motion is the cooling of the unexposed frontal section, fuelling a recurl of this section. This motion then brings the frontal area into the light's beam, step (iii) and the feedback loop which sustains the oscillatory motion is continued. Through this feedback loop based on self‐shadowing and a shifting center of gravity, the film is observed to rock forwards and backwards without any change to the light source. Computational simulations, similar to a previous publication^[15]^ with the addition of solving the heat transfer in the film, show that based on localized heating/cooling and respective bending/unbending of the film extremities, oscillatory motion is expected, see Movie S2. These results support the suggested mechanism derived from experimental observations.

**Figure 4:**
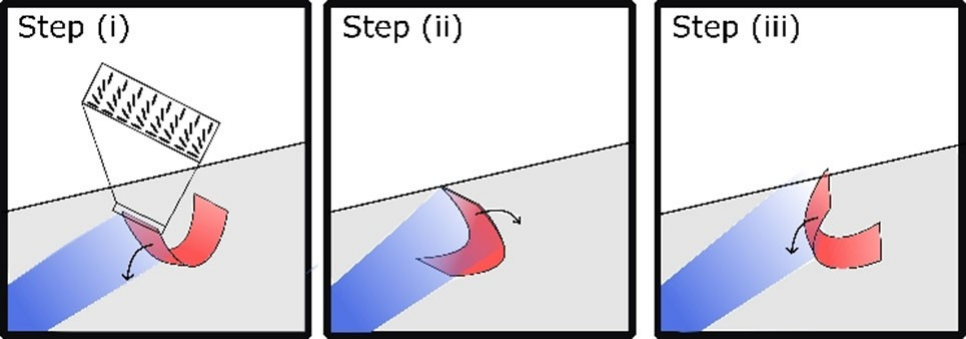
Mechanism for the feedback loop mechanism which fuels the oscillatory motion of the MR‐containing film. When light is incident on the front area of the curled film, light absorption causes the film to unbend towards the light, step (i). This motion shifts the film's center of gravity, causing it to rock towards the light, exposing the rear area of the film while the now unexposed frontal area is brought out of the light beam, step (ii). This then leads to the rear section to unbend towards the surface (away from the light), once again shifting the center of gravity and rocking the film away from the light and bringing the frontal area once again into the light's beam, step (iii). This loop involving actuation and self‐shadowing is continued and fuels the oscillatory motion.

With knowledge of the oscillation mechanism, it is possible to suggest the cause for the intensity dependent variation in oscillation frequency observed in Figure 2C. It is evident that a high oscillation frequency is not directly dependent on the magnitude of the light intensity, as oscillations with a light intensity of 230 mW/cm^2^ are higher than that at 190 mW/cm^2^ or 275 mW/cm^2^. To sustain oscillations, a delicate balance must exist between sufficient actuation to cause the initial tipping of the film (step (i) in Figure 4) and a fast cooling of the shadowed section to fuel a counter motion, step (ii). We suggest intensities around 230 mW/cm^2^ present an optimized situation in which actuation is not only sufficient to cause rapid unbending of the exposed areas of the film,^[21]^ but also allow for the unexposed areas to rapidly cool to a temperature at which the film is curled. With both these aspects, these intermediate intensities allow for films to rapidly rock between two extreme states.

Additionally, considering that the mechanism for oscillations in rooted in the immediate return to the rest, curled state once an area of the films is no longer illuminated, we can also establish the requirement for photothermal dyes. Photomechanical azobenzene dyes do not immediately fuel the return to the film's initial deformation once illumination ceases,^[20]^ but require a secondary illumination step with a different wavelength of light to trigger a return to the photoswitch's ground state.

In summary, this report presents a free standing LCP that sustains light powered oscillatory motion without requiring on/off switching of the light source. The U shaped film oscillates between two extreme states, towards and away from the light, in a manner that resembles the motion of a rocking chair. We demonstrate the motion dependence on the films light absorbance, with high light absorbance being a prerequisite to initiate and sustain oscillations. For oscillatory motion to take place in highly light absorbing films, the necessity for a threshold intensity is demonstrated. Finally, a mechanism for the rocking motion is suggested, being rooted in a feedback loop powered by light‐triggered actuation, self‐shadowing and a shifting center of gravity. The presented actuator further expands the diversity in the possible motions attained with LCPs, contributing to the expansion towards soft robots able to harness sustained motion to achieve greater autonomy.

## Conflict of interest

The authors declare no conflict of interest.

## Supporting information

As a service to our authors and readers, this journal provides supporting information supplied by the authors. Such materials are peer reviewed and may be re‐organized for online delivery, but are not copy‐edited or typeset. Technical support issues arising from supporting information (other than missing files) should be addressed to the authors.

Supplementary

Supplementary

Supplementary
