# Supplementary material for: Light‐Driven Continual Oscillatory Rocking of a Polymer Film
Source: ChemistryOpen. 2020 Nov 6;9(11):1149–52. doi: 10.1002/open.202000237 (PMC7646255; doi:10.1002/open.202000237)
Supplement: Supplementary file 1 — Supplementary [file OPEN-9-1149-s001.pdf]

ChemistryOpen

Supporting Information

Light-Driven Continual Oscillatory Rocking of a Polymer Film

Marina Pilz da Cunha, Akhil R. Peeketi, Adithya Ramgopal, Ratna K. Annabattula, and Albert P. H. J. Schenning*

Supporting Information:

Fabrication of liquid crystalline network

The liquid crystal polymer network is produced from two liquid crystalline monomers: a monoacrylate, 2, (RM 23; 40,5mol%, Merck) and a diacrylate, 1, (RM82; 56,5mol%, Merck), initiated by a photoinitiator (Irgacure 819, 1mol%, Ciba). Light responsivity was achieved with the addition of a commercially available azobenzene chromophore (2 mol%) with a fast cis-trans isomerization, DR, (DR1A, Sigma Aldrich) or with azobenzene derivative, MY, having a longer cis lifetime (Synthon). Prior to polymerization, the monomers were dissolved in dichloromethane (DCM) to obtain a homogeneous mixture; the solvent was subsequently evaporated. Custom-made cells were prepared by gluing together two glass slides coated with differing polyimide alignment layers, (for a splay alignment; one slide with planar and the other with homeotropic alignment layers (Optimer AL 1051 (JSR Micro) and 5661 polyimide (Sunever), respectively)). Glass bead spacers (20 µm diameter) are incorporated into the glue to achieve controlled cell thickness. The cells were filled at 95 °C, at which the LC mixture is isotropic through capillary action. Subsequently, the filled cell was cooled to 80 °C, at which temperature the LC mixture is nematic. Photopolymerization of the reactive mesogens was done at 80 °C with an Exfo Omnicure S2000 lamp; subsequent thermal treatment at 120 °C for 10 minutes released thermal stresses arising from polymer shrinkage during polymerization. After polymerization, the cell was opened, and the films are peeled from the glass with razor blades and cut into the required shapes.

Illumination setup

The LCN was actuated while placed on a surface. Illumination was performed with a LED light source emitting 455 nm (Thorlabs M455L3-C2) mounted with a collimator (ThorLabs SM2F32-1) and driven by a controller (also ThorLabs). The distance between the LED source and the focus was approximately 10 cm.
