# Supplementary figures and images for: Light‐Driven Continual Oscillatory Rocking of a Polymer Film
Source: ChemistryOpen. 2020 Nov 6;9(11):1149–52. doi: 10.1002/open.202000237 (PMC7646255; doi:10.1002/open.202000237)

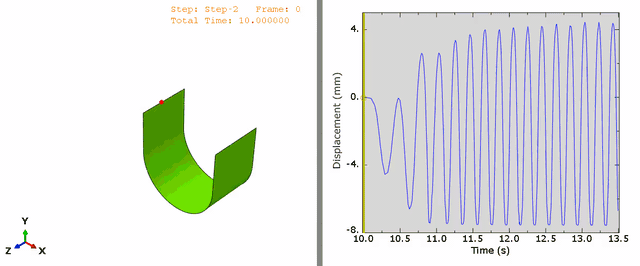

Supplement: Supplementary file 3 — Supplementary [file OPEN-9-1149-s003.gif]
